# CRISPR/Cas9-Mediated Whole Genomic Wide Knockout Screening Identifies Specific Genes Associated With PM_2.5_-Induced Mineral Absorption in Liver Toxicity

**DOI:** 10.3389/fbioe.2021.669434

**Published:** 2021-07-07

**Authors:** Jinfu Peng, Bin Yi, Mengyao Wang, Jieqiong Tan, Zhijun Huang

**Affiliations:** ^1^Center for Clinical Pharmacology, The Third Xiangya Hospital, Central South University, Changsha, China; ^2^Department of Pharmacy, The Third Xiangya Hospital, Central South University, Changsha, China; ^3^Department of Nephrology, The Third Xiangya Hospital, Central South University, Changsha, China; ^4^Center for Medical Genetics, Life Science School, Central South University, Changsha, China

**Keywords:** PM_2.5_, CRISPR/Cas9, liver, mineral absorption, apoptosis

## Abstract

PM_2.5_, also known as fine particles, refers to particulate matter with a dynamic diameter of ≦2.5 μm in air pollutants, that carries metals (Zn, Co, Cd) which can pass through the alveolar epithelium and enter the circulatory system and tissues. PM_2.5_ can cause serious health problems, such as non-alcoholic fatty liver and hepatocellular carcinoma, although the underlying mechanisms of its toxic effect are poorly understood. Here, we exposed L02 cells to PM_2.5_ and performed a pooled genome−wide clustered regularly interspaced short palindromic repeats/CRISPR-associated protein 9 (CRISPR/Cas9) to assess loss of function and identify new potential PM_2.5_targets. Enrichr and KEGG pathway analyses were performed to identify candidate genes associated with PM_2.5_ toxicity. Results revealed that four key genes, namely ATPase Na+/K+ transporting subunit alpha 2 (ATP1A2), metallothionein 1M (MT1M), solute carrier family 6 members 19 (SLC6A19) and transient receptor potential cation channel subfamily V member 6 (TRPV6) were associated with PM_2.5_ toxicity, mainly in regulating the mineral absorption pathway. Downregulating these genes increased cell viability and attenuated apoptosis in cells exposed to PM_2.5_. Conversely, overexpressing TRPV6 exacerbated cell apoptosis caused by PM_2.5_, while a reactive oxygen species (ROS) inhibitor N-acetyl-l-cysteine (NAC) alleviated PM_2.5_-induced apoptosis. In conclusion, ATP1A2, MT1M, SLC6A19 and TRPV6 may be contributing to absorption of metals in PM_2.5_ thereby inducing apoptosis mediated by ROS. Therefore, they hold potential as therapeutic targets for PM_2.5_-related diseases.

## Introduction

Particulate Matter (PM) refers to a suspended mixture of solid and liquid particles in the air (Oh et al., 2021), whereas inhalable fine particles are described by PM_2.5_, usually 2.5 microns or less in diameter (Xing et al., 2016). These potentially harbors various toxic substances, including heavy metals or minerals such as copper (Cu), zinc (Zn), calcium (Ca), potassium (K), and cadmium (Cd) (Ye et al., 2018). These substances can pass through the nasal cavity, enter into the respiratory tract via the airflow and accumulate in tissues through diffusion or active transport (Xing et al., 2016). previous studies have shown that PM_2.5_ is associated with various respiratory disorders (Xing et al., 2016), as well as cardiovascular (Rajagopalan et al., 2018), neurodegenerative (Zhu et al., 2020), and hepatic (Tarantino et al., 2013) diseases. For instance, occurrence of nonalcoholic fatty liver disease – a known silent disease attacking about 20–30% of the population- was linked to exposure to PM_2.5_ (Sivell, 2019). Furthermore, people living in environments with high PM_2.5_ concentrations generally exhibit high incidence of hepatocellular carcinoma (HCC), with an associated high mortality rate (VoPham et al., 2018; Lee et al., 2019).

Previous studies have described the mechanism of PM_2.5_-induced liver disease, with ROS and lysosome implicated in PM_2.5_-induced cell apoptosis (Dornhof et al., 2017; Jing Piao et al., 2018; Zhu et al., 2018). Moreover, PM_2.5_ was shown to induce oxidative stress and inflammation in hepatocytes by altering the normal lipid metabolism (Xu M. X. et al., 2019), farnesin X receptor (FXR) (Wang et al., 2020), ROS/PINK1/Parking Signal pathways causing NADPH oxidation, and liver fibrosis (Zheng et al., 2015; Qiu et al., 2019). To date, however, the underlying mechanism of PM_2.5_ -mediated hepatotoxicity remains unclear, necessitating further exploration.

Clustered regularly interspaced short palindromic repeats/CRISPR-associated protein 9 (CRISPR/Cas9), an RNA-guided DNA endonuclease, can be easily programmed to target new sites by changing its guide RNA sequence (Shalem et al., 2014; Wang et al., 2016). CRISPR/Cas9 is a potent gene-editing tool, that enables direct and accurate editing of DNA (Lino et al., 2018). This technology can be experimentally be applied to evaluate and modify the functions of thousands of genes, identify and verify new drug targets and detect potential diseases in humans (Huang A. et al., 2020). CRISPR/Cas9 has also been applied in exploration of pathogenesis of the nonalcoholic fatty liver disease, affirming its role as a promising method for genetic engineering of liver cancer (Ratan et al., 2018; Gordon et al., 2019). Here, we applied genome-wide CRISPR/Cas9 (GeCKO) technology to unravel new regulatory factors associated with PM_2.5_ toxicity in human cell line L02. The method also revealed some drug-resistant genes in response to PM_2.5_, which may be utilized as potential therapeutic targets.

## Materials and Methods

### Lentiviral Production of the Single Guide RNA Library

**FIGURE 1:**
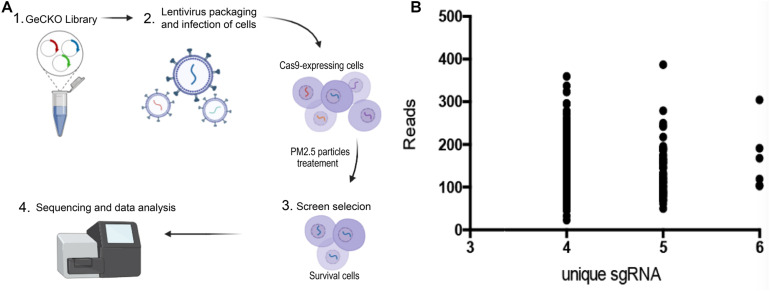
Genome-wide CRISPR/Cas9-mediated forward genetic screening for identification of genes associated with PM2.5 resistance. **(A)** Schematic representation of forwarding genetic screening in L02 cells based on pooled sgRNA library. **(B)** PM_2.5_ resistance genes identified after screening. Number of unique sgRNAs genes (X-axis) and changes in the number of reads of each sgRNA relative to controls (Y-axis).

We generated lentiviruses using a previously described protocol (Cai et al., 2019). Briefly, a day before transfection, HEK293T cells (ATCC, United States) were cultured in DMEM (Gibco) containing 10% fetal bovine serum (FBS, Invitrogen, United States) and maintained at 37°C (5% CO_2_). The fusion rate was about 50%. The cells were transfected with 4 μg LentiCRISPR plasmid library (#1000000048, Addgene), 2 μg of pVSVg (#8454, Addgene) and 6 ug psPAX2 (#12260, Addgene) in a petri dish at 10 cm^2^ using Lipofectamine 2000 (Invitrogen, United States), according to the manufacturer’s instructions. After 48 h of culture, the contents were transferred into a test tube, then cell fragments precipitated via centrifugation at 3,000 rpm for 10 min. The supernatant was filtered (with a pore size of 0.45 μm), and ultracentrifuged for 2 h at 24,000 rpm at 4°C. Finally, the virus preparation was suspended in DMEM at 4°C overnight and stored at −80°C after being divided equally (Figure 1A, step 1).

### Lentiviral Transduction of the sgRNA Library

**FIGURE 2:**
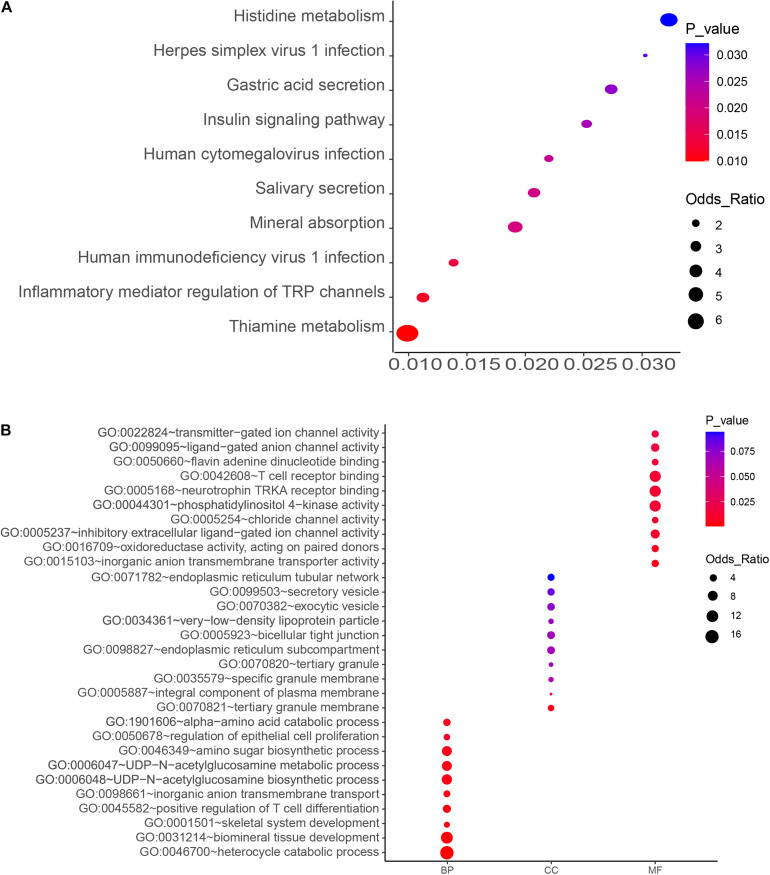
KEGG pathway **(A)** and GO enrichment **(B)** analyses of the top-ranked PM_2.5_ resistance genes with high number (4–6) of unique sgRNAs. Significant processes and pathways are depicted in red. BP, GO Biological Process; MF, GO Molecular Function; CC, GO Cellular Component).

L02 cells were purchased from ATCC, and cultured in DMEM (Gibco), supplemented with FBS 10% (Invitrogen, United States). Gecko library was used to infect 3 × 10^8^ cells. The multiplicity of infection (MOI) was 0.1, and aimed to ensure that most cells received only 1 viral construct. The culture medium containing cells was supplemented with 10% FBS and 4 mM l- glutamic acid (Invitrogen), 10 μg/ml penicillin and streptomycin (Invitrogen, United States), followed by addition of the lentivirus to each dish with 8 μg/mL polybrene (Sigma). The cultures were incubated for 48 h, medium aspirated out, replaced with fresh DMEM supplemented with 1 μg/ml doxycycline, followed by incubation for 7 days. The cell population was created, with each target gene theoretically carrying a mutation of functional loss (Figure 1A, 2) (Wang et al., 2014b).

### Screening for PM_2.5_ Resistance Genes *via* DNA Sequencing

Cells were exposed to PM_2.5_ (0.1 mg/mL), purchased from the National Institute of Standardization and Technology (1648a, Urban Particles), for 48 h, followed by transfer to a glucose-containing medium (95% air, 5% CO_2_) for 6-h recovery. After exposure to PM_2.5_, the cells were merged for sgRNA analysis (Figure 1A, step 2–3). Genomic DNA was extracted from living cells using the DNA Extract All Reagents Kit (Invitrogen, United States) and stored it at −20°C. The DNA was used for PCR amplification, using the following primers; Forward primer: CTTGTGGAAAGGACGAAACA; and Reverse primer: GCCAATTCCCACTCCTTTCA. The PCR conditions are 95°C for 5min, 54°C for annealing, 72°C for 30 s, 35 cycles. PCR amplicons were sequenced on the HiSeq 2500 platform (Illumina) as previously described (Shalem et al., 2014).

Sequencing of sgRNAs amplified from the genome of surviving cells was done using Next-generation sequencing (NGS), and the number of unique sgRNAs and NGS reads used to classify candidate genes. We adopted a customized CRISPR-Cas9 library screening pipeline to process and analyze raw sequence data. Briefly, the barcode in the reverse primer was first used to analyze the repeated sequential readings, then Cutadapt used to delete the sequence from the starting point of sgRNA. The sgRNA sequence was mapped to the pooled GeCKO v2 library A and B, using the trimmed reads, and read counts from all samples quantified by MAGeCK 5. 6. 0. Counting data were filtered and standardized, then essential sgRNA and genes ranked using MAGeCK. Unique sgRNAs with a high number (4–6) were defined as top-level genes (Figure 1A, 4).

### KEGG Pathway and GO Analysis of PM_2.5_ Resistance Genes

KEGG pathway analysis and Gene Ontology (GO) were performed for functional annotation enrichment analysis of top-level genes with a high number (4–6) of unique sgRNAs using Enrichr^1^ (Kuleshov et al., 2016). Gene Ontology function annotation comprised three categories, namely biological processes (BP), cell components (CC) and molecular functions (MF). Gene Ontology terms and KEGG pathways were downloaded from the website, at a threshold of *P*-value < 0.05.

### Cell Cultures, RNA Silencing, and TRPV6 Overexpression

L02 cells were seeded on 24-well plates and cultured in DMEM (Gibco) containing FBS 10% (Invitrogen, United States) and maintained at 37°C in a humidified incubator (5% CO_2_). Cells were divided into six groups: siMTM1, siTRPV6, siATP1A2, siATP1B2, siSLC6A19, and control siRNA. Small interfering RNAs (siRNAs) was bought from GenePharma (Shanghai, China). siRNAs and their corresponding control RNAs were transfected using Lipofectamine 2000 (Invitrogen, United States) according to the manufacturer’s instructions. Exactly 50 pmol of siRNAs/well were transfected into cells in a 24-well plate with 1 μL Lipofectamine 2000. The cells were exposed to PM_2.5_ (0.1 mg/ml) for 48 h, then incubated for different periods under conditions of 95% air, 5% CO_2_, and glucose-containing medium to induce apoptosis. Negative controls (NC, Control) were incubated with medium (FBS 10%) without PM_2.5_.

TRPV6 (NM_018646) plasmid or negative control plasmid (purchased from Origene [#RC214982]) was transfected into L02 cells: TRPV overexpression cells or control, then divided into six groups: negative control, TRPV6, PM_2.5_ treated negative control or TRPV6, PM_2.5_ and NAC (N-acetyl-l-cysteine) treated negative control or TRPV6. The cells, transfected by plasmid, were exposed to PM_2.5_(0.1 mg/ml) for 48 h, followed by different periods of 95% air, 5% CO_2,_ and glucose-containing medium to induce apoptosis. Negative controls (NC, Control) were incubated with medium (FBS 10%) without PM_2.5_. We design experiment with three replicates, each replicate has like three samples for every groups.

### MTT Assay

Cell viability was assessed using colorimeter 3,4,5-dimethylthiazole -2- yl -2,5- diphenyltetrazolium bromide (MTT). Briefly, 30 μL of MTT solution was added to each well containing cells, incubated at 37°C for 3 h, the medium aspirated out and dried overnight. The following day, the formazan crystal was dissolved in 50 μL of dimethyl sulfoxide (DMSO), mixed on a shaker for 1 h, readings taken on a spectrophotometer at 570 nm wavelength, followed by analysis of percentage cell activity.

### Analysis of Cell Apoptosis

Cell apoptosis was assessed via Annexin V-FITC/Propidium Iodide (PI) double staining, using the cell apoptosis detection kit (Nanjing KeyGen Biotech Co., Ltd.). Briefly, L02 cells were collected and washed twice with PBS, then mixed with 500 μL binding buffer, 5 μL annexin V-FITC, and 10 μL polyimide. the cultures were incubated at 37°C for 10 min in the dark at, then apoptosis analyzed via flow cytometry (BD Biosciences, United States) and Cell QuestPro software (BD Biosciences).

### Quantification of Apoptotic Nuclei by DAPI Staining

Apoptotic nuclei were quantified through DAPI Staining. Briefly, cells were first washed with PBS, fixed in 40% paraformaldehyde (20 min) and permeabilized in 0.1% (w/v) Triton X100 (15 min). The cells were stained with DAPI (4′,6-diamidino-2-phenylindole) for 15 min, washed with PBS and examined under a fluorescent microscope (Leica, Germany).

### ROS Detection

Production of ROS in cells was measured by oxidation of cell-permeable dyes DCFDA/H2DCFDA – Cellular ROS Assay Kit (ab113851, Abcam, United States) following the manufacturer’s instructions. Briefly, cells were digested with collagenase IV (Gibco), precipitated and suspended in a medium containing 20 μM DCFDA, with a 30-min incubation. The cells were centrifuged at 2,000 rpm for 10 min, suspended in a fresh medium, then analyzed via flow cytometry (BD Biosciences, United States).

### Activation of Lysosomal Function in Cells

The intralysosomal pH was estimated using LysoSensor^TM^ Green DND-189 (# L7535, Invitrogen, USA). Briefly, cells were incubated with 5 μM hemolysis sensor green DND-189 in DMEM at 37°C for 5 min, and their nuclei examined using DAPI. Intensity of fluorescence was examined, images of typical cells taken using a confocal microscope (Leica TCS SP8, Leica Microsystems). A cell-lysate-based assay for cathepsin B/D activity was performed using Cathepsin B kit (Abcam Plc. Cambridge), according to the manufacturer’s protocol. Summarily, cells were lysed in a lysis buffer, then the lysate incubated, for 1 h, with 50 μM fluorescent cathepsin B substrate (Z-RR-AMC) in a cell-free system containing buffer (10 mM HEPES-NaOH, pH 7.4) in a plate at 37°C. Fluorescence intensity was monitored at 400 and 505 nm wavelengths using a fluorometer (ThermoFisher Scientific Inc), and the data compared between treated and control groups.

### Statistical Analysis

All statistical analyses were performed using Graphpad Prism 6 software, and the data presented as means ± standard errors of the mean (SEM). Comparisons between and among groups were performed using a student’s *t*-test and one-way analysis of variance (ANOVA), respectively, at a significance level of *p* ≤ 0.05.

## Results

### Genome-Wide CRISPR/Cas9−Mediated Screening Identifies PM_2.5_ Resistance Genes

A GeCKO library, containing 123,411 sgRNA for 19,050 human genes, was inserted into the lentiviral vector to generate a pool of cells with targeted genes carrying a loss−of−function mutation. GeCKO detection revealed that cells exposed to PM_2.5_ were enriched in multiple sgRNAs. We deduced that the loss of homologous genes confers resistance to PM_2.5_. A scatter plot of sgRNA number and corresponding sequence reads (Figure 1B), showed that the detected genes were well-distributed in every sgRNA. A total of 614 top-level genes, with a high number (4–6) of unique sgRNAs among the 19,050 genes, were identified and these were associated with toxic effects of PM_2.5_ (Supplementary Table 1). We also identified some interesting gene defects that were potentially associated with protection from PM_2.5_ toxicity (Supplementary Table 2). Generally, these genes were involved in mineral absorption (ATP1A2, ATP1B2, MT1M, SLC6A19, and TRPV6) and inflammatory mediators regulating TRP channels (ADCY9, ASIC5, calm5, CYP4A22, CALML4, IL1RAP, PIK3R1, and PLCG1).

### KEGG Pathway and GO Analysis of PM_2.5_ Resistance Genes

The top 10 key KEGG pathways and GO functions are illustrated in Figure 2. Specifically, KEGG analysis identified 17 significant pathways (*P* < 0.05) (Supplementary Table 3). Particularly, the mineral absorption pathway was significantly associated with a group of genes, namely ATP1A2, SLC6A19, MT1M, TRPV6, and ATP1B2) (*p* = 0.019) (Figure 2 and Supplementary Table 2) whereas inflammatory mediator regulation of TRP channels comprised ADCY9, ASIC5, CALML5, CYP4A22, CALML4, IL1RAP, PIK3R1, and PLCG1. Gene Ontology analysis revealed several biological processes associated with PM_2.5_, including biological mineral tissue development (*p* = 0.0014), skeletal system development (*p* = 0.0018), and inorganic anion transport across membrane (*p* = 0.0033). On the other hand, those significantly associated with molecular function included inorganic anion transmembrane transporter activity (*p* = 0.0033), inhibitory extracellular ligand-gated ion channel activity (*p* = 0.0099), chloride channel activity (*p* = 0.01), and transmitter-gated ion channel activity (*p* = 0.017). With regards to cellular components, we identified tertiary granular membrane (*p* = 0.0069), specific granular membrane (*p* = 0.06), and tight connection between two cells (*p* = 0.069) as the significant GO terms (Figure 2 and Supplementary Table 3). Apart from these, antioxidant enzyme activity (GO: 0016709) and T cell receptor binding (GO: 0042608) were also identified, suggesting a possible association with PM_2.5_-induced oxidative stress and inflammation.

### Mineral Absorption-Related Genes Affect PM_2.5_−Induced Apoptosis

**FIGURE 3:**
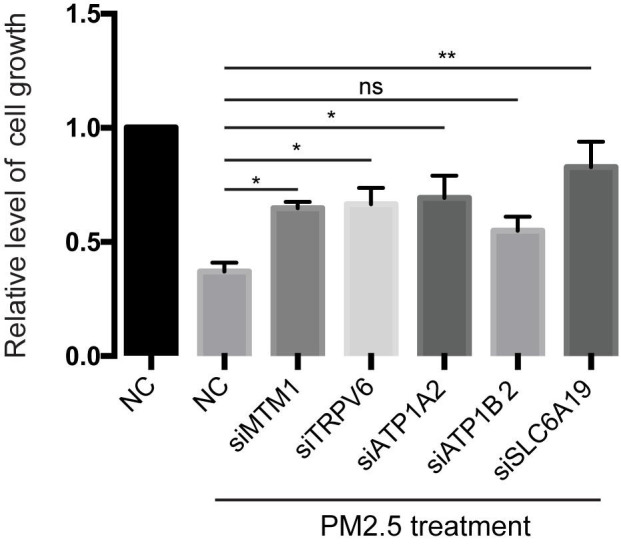
Knockdown of mineral absorption-related genes rescued the reduction of cell viability by PM_2.5_. L02 cells were transfected with siRNAs targeting each candidate gene, then cells exposed to PM_2.5_. Cell viability was analyzed by MTT. *p < 0.05, **p < 0.01. ns, no significance. Data are representative of three independent experiments with *n* = 3 (Mean ± SEM), normalized by NC group.

**FIGURE 4:**
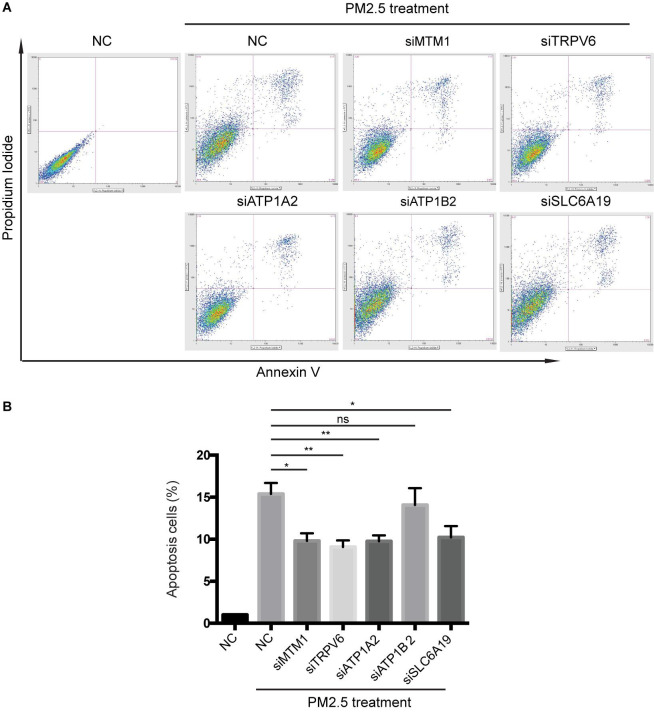
Knocking out of genes associated with mineral absorption suppressed PM_2.5_-induced apoptosis. **(A)** Cells were treated with PM_2.5_, while apoptosis was detected via Annexin V + PI staining. NC, Negative control. **(B)** Analysis of relative levels of cell apoptosis. *p < 0.05, **p < 0.01. ns, no significance. Data are representative of three independent experiments with *n* = 3 (Mean ± SEM), normalized by NC group.

Knocking out ATP1A2, MT1M, SLC6A19, or TRPV6 resulted in significant elevation of cell survival rate following PM_2.5_ exposure (Figure 3). On the other hand, knocking out ATP1A2, ATP1B2, MT1M, SLC6A19, or TRPV6 potentially suppressed the rate of apoptosis following PM_2.5_ exposure (Figure 4). These findings indicate that these genes might enhance sensitivity of cells exposed to PM_2.5_ to apoptosis.

### Metals Absorbed by TRPV6 Cause PM_2.5_-Induced Apoptosis Through ROS Production

**FIGURE 5:**
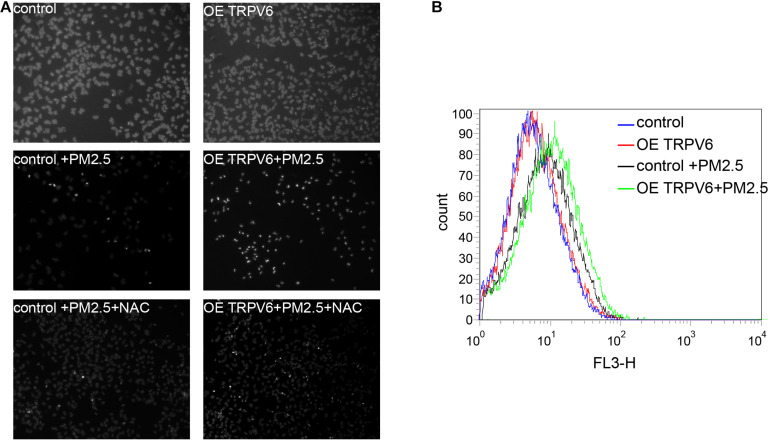
Metal absorption by TRPV6 initiated ROS-mediated PM_2.5_-induced apoptosis. **(A)** Fluorescent images indicate the degree of apoptosis. DAPI was used to stain the nucleus to evaluate apoptosis, nuclear pyknosis (bright, small, irregular) represented apoptosis. Normal L02 cells and TRPV6 overexpressing L02 cells showed good growth (upper panel). PM_2.5_-induced apoptosis of L02 cells (middle left, fluorescent represents apoptotic cells), and L02 cells overexpressing TRPV6 showed worse growth (middle right). Addition of NAC suppressed the effect of PM_2.5_, and upregulated TRPV6 (lower panel). TRPV6 overexpression increased apoptosis of L02 cells, whereas NAC (ROS inhibitor) decreased apoptosis. **(B)** Evaluation of ROS production in cells using DCFDA. ROS was evaluated by staining cells with a DCFDA cell reactive oxygen species detection kit. ROS levels were quantified via flow cytometry (BD Biosciences, United States). PM_2.5_ (black line) induced ROS generation, while TRPV6 overexpression (green line) elevated the effect of PM_2.5_.

Results above indicated that TRPV6 exerted the most significant role in PM_2.5_-induced apoptosis. Therefore, we selected TRPV6 for further exploration of PM_2.5_-induced apoptosis. Rescue experiments revealed that overexpressing TRPV6 could aggravate PM_2.5_-induced apoptosis, whereas N-acetyl-l-cysteine (NAC), a ROS inhibitor potentially blocked PM_2.5_- and TRPV6-induced apoptosis (Figure 5A). Besides, overexpressing TRPV6 positively promoted ROS production (Figure 5B). These findings suggest that TPRV6 promotes the toxic effects of PM_2.5_, thereby aggravating ROS production in the cell, leading to apoptosis. Besides, we found no evidence of activation of lysosomal cell function after PM_2.5_ exposure (Supplementary Figure 1), indicating that the lysosome did not mediate PM_2.5_-induced apoptosis.

## Discussion

PM_2.5_ particles have been implicated in occurrence of diseases, such as nonalcoholic fatty liver disease (Tarantino et al., 2013) and HCC (VoPham et al., 2018). Notably, these disease have been associated with PM_2._5-induced apoptosis, which is one of the primary pathological characteristics (Peng et al., 2017; Huang X. et al., 2020). CRISPR screening represents a key approach for identifying essential genes or genetic sequences that trigger specific functions or phenotypes. In the present study, this method allowed us to identify 19,050 genes associated with PM_2.5_ resistance. Particularly, GeCKO screening revealed 614 genes that included a large number (4–6) of unique sgRNAs, potentially related to PM_2.5_ toxicity. Functional analyses showed that the PM_2.5_ resistant genes were associated with absorption of liver minerals and regulation of inflammatory environments, suggesting that they may be playing a crucial role in apoptosis of hepatocytes.

**FIGURE 6:**
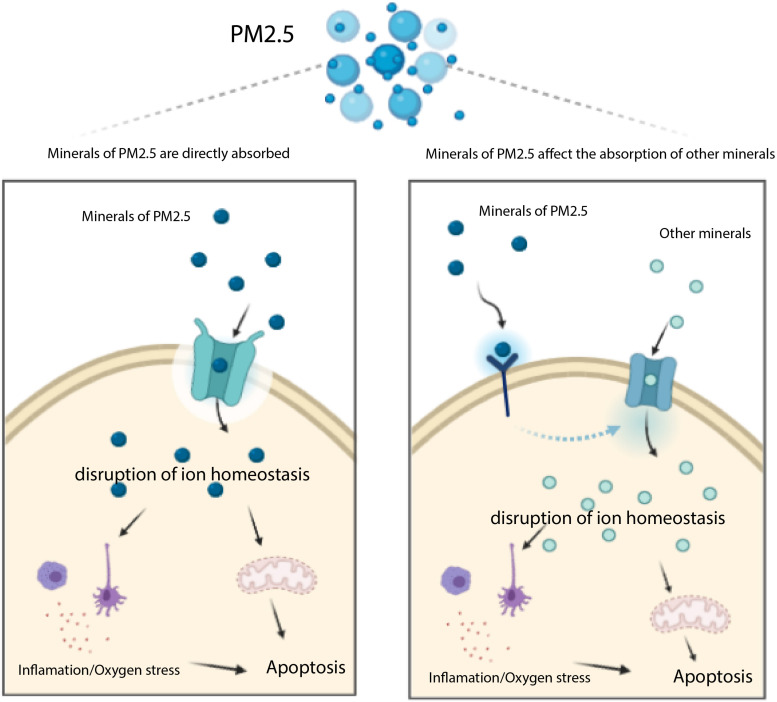
Transportation of toxic chemicals (metals and minerals) in PM2.5 induces apoptosis mediated by ROS. The minerals of PM2.5 may be trasported into cell through transporters (e.g. MT1M, ATP1A2, SLC6A19, and TRPV6 encoding proteins), they may also affect other transporters which uptake other minerals inducing apoptosis.

Previous studies have shown that major heavy metals of PM_2.5_ potentially accumulate in the liver (Li et al., 2015), whereas genes associated with transportation of toxic chemicals (metals and minerals) in PM_2.5_ may play an important role in apoptosis and damage of liver cells (Figure 6). Notably, our KEGG analysis revealed several pathways associated with PM_2.5_ resistance, namely the insulin signaling pathway, phosphatidylinositol signaling system, adrenergic signaling in cardiomyocytes, gastric acid secretion, and inflammatory mediator regulation of TRP channels, consistent with previous studies (Xu Z. et al., 2019; Sanchez et al., 2020; Zheng et al., 2020). One of the most significant pathway was that regulating mineral absorption, which comprised several genes including ATP1A2, SLC6A19, MT1M, TRPV6, and ATP1B2, indicating that the absorption of metals by the liver is essential for PM_2.5_ toxicity. Notably, ATP1A2, ATP1B2, MT1M, SLC6A19, and TRPV6 contribute to absorption of metals and minerals. Moreover, downregulating these genes suppressed accumulation of metals and minerals in L02 cells after PM_2.5_ exposure. On the other hand, regulation of inflammatory media in the TRP channels was also significantly enriched, and comprised ADCY9, ASIC5, calm5, CYP4A22, CALML4, IL1RAP, PIK3R1, PLCG1 genes following exposure to PM_2.5_. This implied that PM_2.5_ exposure induced oxidative stress and cellular lesions, in line with a previous study that found PM_2.5_ to be the primary cause of oxidative stress and inflammation in hepatocytes (Xu M. X. et al., 2019).

Gene Ontology (GO) analysis revealed that PM_2.5_ resistance genes were mainly associated with bio-mineral tissues and skeletal systems, such as biological mineral tissue and skeletal system development. Previous studies have shown that minerals play a crucial role in development of bio-mineral tissues and skeletal systems (Office of the Surgeon General, 2004; Upadhyay, 2017), indicating that genes related to minerals play a role in PM_2.5_ resistance. Other GO functions identified in the present study included inorganic anion transport across membrane, inhibitory extracellular ligand gated ion channel activity, chloride channel activity and transmitter gated ion channel activity, among others, which are also indirectly associated with the inter-cellular transfer of ions and minerals (or metals). Furthermore, oxidative stress and inflammation were the consequence of the absorption of metal ions in PM_2.5_. Herein, we found that the antioxidant enzyme activity (GO: 0016709) and T cell receptor binding (GO: 0042608) may amount to PM_2.5_-induced oxidative stress and inflammation (Cope, 2003; Deleonardi et al., 2004). Overall, GeCKO screening revealed that the PM_2.5_ resistance genes identified in L02 cells were associated with the mineral absorption and inflammation-associated pathways, while Go terms confirmed that the PM_2.5_ resistance genes play a key role in mineral absorption, inflammation, and induction of oxidative stress.

Overexpressing siRNAs confirmed that MT1M, ATP1A2, SLC6A19, and TRPV6 genes were associated with mineral absorption and protection of liver cells from apoptosis. The Certificate of Analysis of Standard Reference Material^®^ 1648a listed 25 metals in PM_2.5_, including Cd, Cu, Zn, Ca, K, and Na. Previous studies have shown that MT1M is a vital component of the metallothionein (MTs) family, which comprises cysteine and short peptide of thioprotein with a high affinity for heavy metals such as Cd, Zn, Cu. Functionally, MR1M plays an indispensable role in homeostasis and detoxification of metal ions (Si and Lang, 2018), whereas TRPV6 is a member of the TRP ion channel family with the highest affinity for Ca^2+^ and mainly functions in absorption of Ca^2+^ in the intestinal tract. The intervention to TRPV6 can alter calcium absorption and bone mineralization and the early stages of epithelial cell hyperplasia and malignancy (Holzer, 2011). On the other hand, ATP1A2 encodes the α2 subtype of the Na^+^, K^+^-ATPase’s catalytic subunit, and functions as an ion channel/ion transporter, while SLC6A19 encodes an amino acid delivery system B (0) AT1, which mediates transfer of neutral amino acids to the intracellular space from the luminal compartment (Cheon et al., 2010). Results of the present study showed that overexpressing TRPV6 elevated apoptosis, but knockdown of ATP1A2, SLC6A19, MT1M, and TRPV6 significantly improved the rate of cell survival and suppressed apoptosis. Particularly, MT1M, ATP1A2, SLC6A19, and TRPV6 facilitated transportation of metals and minerals to cells, which was closely related to the toxic effects of PM_2.5_.

Previous studies have shown that PM_2.5_ induces oxidative stress and inflammation, thereby indirectly initiating apoptosis and disease development (Jomova and Valko, 2011; Jian et al., 2018; Qiu et al., 2019; Xu M. X. et al., 2019). Our results corroborated these findings, in that metals in PM_2.5_ might play a highly crucial role in this process. Numerous studies have reported that metals in PM_2.5_, such as Zn, Co, Cd, and Au, can induce cell death by activating ROS production (Sharma et al., 2012; Trejo-Solís et al., 2012; Wang et al., 2014a; Hu et al., 2015; Iranpak et al., 2019). Results of the present study showed that NAC (ROS inhibitor) suppressed apoptosis induced by metals absorbed by TRPV6. GeCKO screening further revealed absence of particular genes associated with inflammation and oxidoreductase activity, including IL1RAP (Wood et al., 2009), PIK3R1, PLCG1 (Jiang et al., 2018), CYP26B1, CYP4F11 (Jian et al., 2018), and MICAL2 (Mariotti et al., 2016). A previous study reported that accumulation of intracellular PM_2.5_ promoted lysosomal destabilization and cell death (Dornhof et al., 2017). In the present study, we found no evidence that lysosome mediated PM_2.5_-induced apoptosis, although further studies are needed to explore the relationship between lysosomal destabilization and cell death, other than apoptosis.

The present study had some limitations. Firstly, we focused on transporter genes, and did not evaluate the concentration and accurate types of metals or minerals. Secondly, although mineral transporters, such as TRPV6, have been shown to be critical for PM_2.5_, particularly in inducing ROS production and cell apoptosis, the underlying mechanism of transport remains unclear, thus necessitating further exploration. Experimental works at our laboratory are expected to clarify the role of metal or mineral transport proteins in ROS-mediated PM_2.5_ toxicity.

## Conclusion

Metals represent the main component of PM_2.5_, and these play a crucial role in PM_2.5_ -induced apoptosis. The distribution of metals into liver cells through the transporter induces apoptosis. Our results identified several genes associated with mineral absorption, including ATP1A2, ATP1B2, MT1M, and TRPV6, and these were also related to ROS-mediated apoptosis, following absorption of metals in PM_2.5_. Overall, these results provide theoretical support for designing strategies for management of injuries caused by PM_2.5_ particles.

## Data Availability Statement

The original contributions presented in the study are publicly available. This data can be found here: http://www.ncbi.nlm.nih.gov/sra/, SRR14361138.

## Author Contributions

ZH: conceptualization and funding acquisition. BY, JT, and MW: methodology and experiments. BY: formal analysis. JP: writing–original draft preparation. JP, BY, MW, JT, and ZH: writing–review and editing. BY, JT, and ZH: supervision. All authors have read and approved the final manuscript.

## Conflict of Interest

The authors declare that the research was conducted in the absence of any commercial or financial relationships that could be construed as a potential conflict of interest.

## Footnotes

1 https://maayanlab.cloud/Enrichr/

## Supplementary Material

Supplementary Figure 1 Activation of lysosomal function in cells in the absence and presence of PM_2.5_. **(A)** L02 and TRPV6 overexpression cells were treated with PM_2.5_. Cells were then stained with LysoSensor^TM^ Green DND-189. There was no difference between groups; **(B)** cathepsin B and cathepsin D activities in lysosome were determined by Cathepsin B Activity Assay Kit. TRPV6 and PM_2.5_ do not affect the activation of lysosomal function in cells.

Supplementary Table 1 Top-rank genes with a high number (4–6) of unique sgRNAs involved in PM_2.5_ toxic effect. Average: Sum divided by count; Count: The unique sgRNAs number of the gene; HGLibA/Bxxx; Code assigned to each sgRNA in the GeCKO Library; sgRNA: Short-guided RNA; sum: Total number of sgRNA readings per gene. Data are representative of three independent experiments with *n* = 3 (Mean ± SEM), normalized by NC group.

Supplementary Table 2 KEGG pathway analysis of higher PM_2.5_ resistance genes. Pathway: The name of the KEGG pathway, Overlap: The ratio of the number of overlapping genes to the number of genes entered, P-value: Use Fisher’s exact test to calculate the P-value, Odds Ratio: The deviation from the expected ranking, Composite score: Multiplying the logarithm of the p-value from the Fish exact test by the deviation z- score from the expected ranking, Gene: Overlapping genes between inputs and paths.

Supplementary Table 3 GO enrichment analysis of the top-rank PM_2.5_ resistance top-rank genes. (Function: GO analysis function name, Overlap: Ratio of overlapping genes to input genes, P values, Odds Ratio, comprehensive scores, and genes: same with legends of Supplementary Table 2).

## Abbreviations

CRISPR: clustered regularly interspaced short palindromic repeats
Cas9: CRISPR-associated protein 9
PM_2.5_: Particulate Matter 2.5
ATP1A2: ATPase Na+/K+ Transporting Subunit Alpha 2
ATP1B2: ATPase Na+/K+ transporting subunit beta 2
MT1M: metallothionein 1M
SLC6A19: Solute Carrier Family 6 Member 19
TRPV6: Transient Receptor Potential Cation Channel Subfamily V Member 6
GeCKO: genome − scale CRISPR/Cas9 knockout
NGS: next − generation sequencing
sgRNA: single guide RNA
IL1RAP: interleukin 1 receptor accessory protein
PIK3R1: phosphoinositide-3-kinase, regulatory subunit 1 (alpha)
PLCG1: phospholipase C gamma 1
MICAL2: Microtubule Associated Monooxygenase, Calponin and LIM Domain Containing 2
NAC: N-acetyl -l-cysteine
ROS: reactive oxygen species
DAPI: 4′,6-diamidino-2-phenylindole
DCFDA: dyes 2′,7′-dichlorodihydrofluorescein diacetate.
