# Supplementary figures and images for: CRISPR/Cas9-Mediated Whole Genomic Wide Knockout Screening Identifies Specific Genes Associated With PM2.5-Induced Mineral Absorption in Liver Toxicity
Source: Front Bioeng Biotechnol. 2021 Jul 7;9:669434. doi: 10.3389/fbioe.2021.669434 (PMC8293916; doi:10.3389/fbioe.2021.669434)

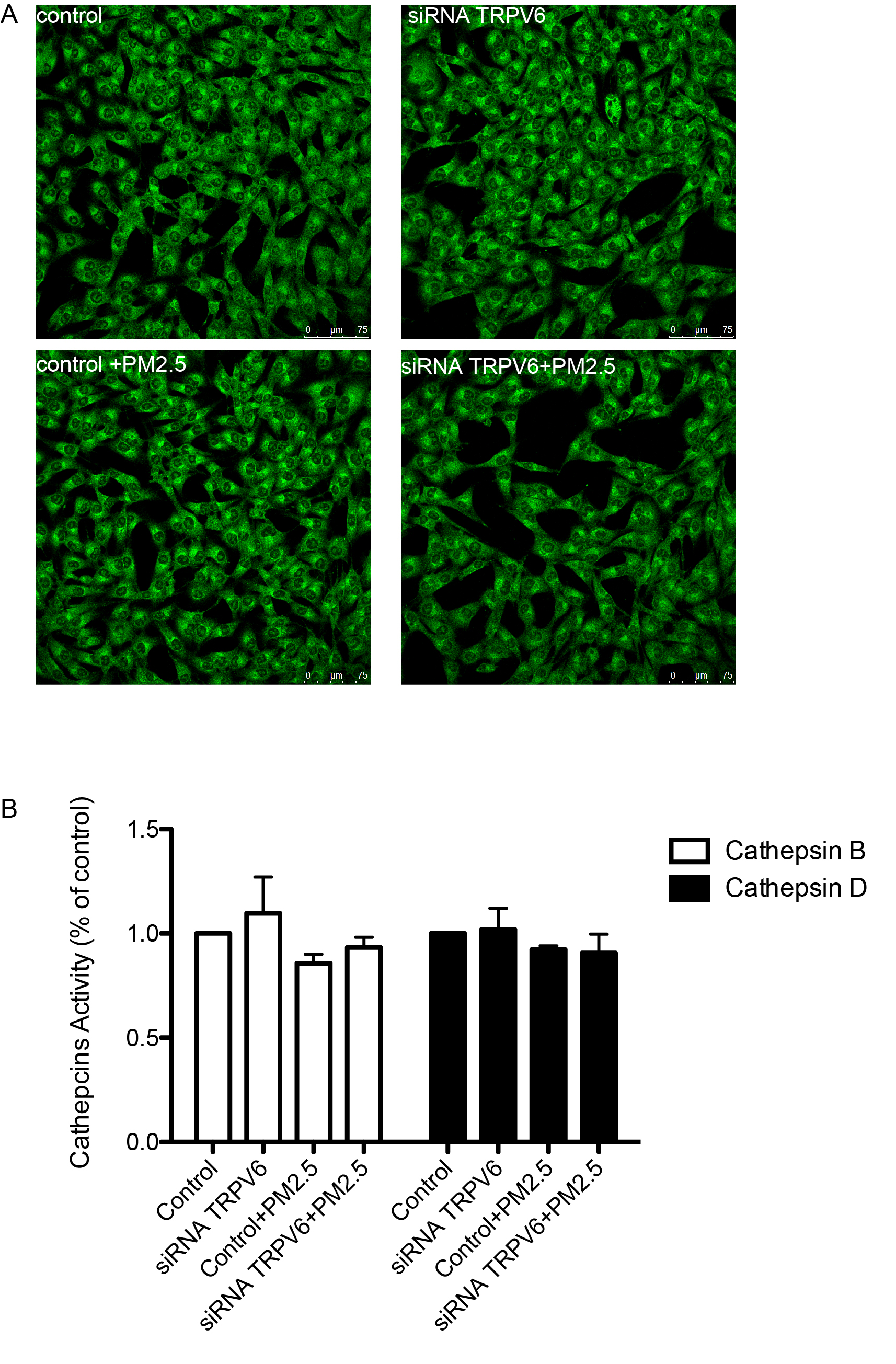

Supplement: Supplementary Figure 1 — Activation of lysosomal function in cells in the absence and presence of PM2.5. (A) L02 and TRPV6 overexpression cells were treated with PM2.5. Cells were then stained with LysoSensorTM Green DND-189. There was no difference between groups; (B) cathepsin B and cathepsin D activities in lysosome were determined by Cathepsin B Activity Assay Kit. TRPV6 and PM2.5 do not affect the activation of lysosomal function in cells. [file Image_1.JPEG]
